# Nosocomial Infection in Adult Admissions with Hematological Malignancies Originating from Different Lineages: A Prospective Observational Study

**DOI:** 10.1371/journal.pone.0113506

**Published:** 2014-11-21

**Authors:** Hui Liu, Jin Zhao, Yubin Xing, Meng Li, Mingmei Du, Jijiang Suo, Yunxi Liu

**Affiliations:** 1 Department of Digestive Medicine, the Second Affiliated Hospital of Dalian Medical University, Dalian, China; 2 Department of Infection Management and Disease Control, Chinese PLA General Hospital, Beijing, China; 3 Department of Respiratory Medicine, Chinese PLA General Hospital, Beijing, China; 4 Department of Hematology, Chinese PLA General Hospital, Beijing, China; Queen's University Belfast, United Kingdom

## Abstract

**Background:**

Nosocomial infection (NI) causes prolonged hospital stays, increased healthcare costs, and higher mortality among patients with hematological malignancies (HM). However, few studies have compared the incidence of NI according to the HM lineage.

**Objective:**

To compare the incidence of NI according to the type of HM lineage, and identify the risk factors for NI.

**Methods:**

This prospective observational study monitored adult patients with HM admitted for >48 hours to the General Hospital of the People's Liberation Army during 2010–2013. Attack rates and incidences of NI were compared, and multivariable logistic regression was used to control for confounding effects.

**Results:**

This study included 6,613 admissions from 1,922 patients. During these admissions, 1,023 acquired 1,136 NI episodes, with an attack rate of 15.47% and incidence of 9.6‰ (95% CI: 9.1–10.2). Higher rates and densities of NIs were observed among myeloid neoplasm (MN) admissions, compared to lymphoid neoplasm (LN) admissions (28.42% vs. 11.00%, P<0.001 and 11.4% vs. 8.4‰, P<0.001). NI attack rates in acute myeloid leukemia (AML) and myelodysplastic/myeloproliferative neoplasm (MDS/MPN) were higher than those in MDS (30.69% vs. 20.19%, P<0.001; 38.89% vs. 20.19%, P = 0.003). Attack rates in T/NK-cell neoplasm and B-cell neoplasm were higher than those in Hodgkin lymphoma (15.04% vs. 3.65%; 10.94% vs. 3.65%, P<0.001). Multivariable regression analysis indicated prolonged hospitalization, presence of central venous catheterization, neutropenia, current stem cell transplant, infection on admission, and old age were independently associated with higher NI incidence. After adjusting for these factors, MN admissions still had a higher risk of infection (odds ratio 1.34, 95% CI: 1.13–1.59, P<0.001).

**Conclusion:**

Different NI attack rates were observed for HM from different lineages, with MN lineages having a higher attack rate and incidence than LN lineages. Special attention should be paid to MN admissions, especially AML and MDS/MPN admissions, to control NI incidence.

## Introduction

Patients with hematological malignancies (HM) are vulnerable to nosocomial infections (NIs), as the underlying diseases and aggressive treatment strategies typically impair the patient's immune system [1]–[3]. NIs can result in longer hospital stays, increased healthcare costs, and even higher mortality [4], [5]. Mortality attributable to bloodstream NIs in cancer patients ranges between 10% and 20%, while mortality from nosocomial pneumonia is much higher (between 40% and 60%) [6]–[10]. The Clinical Advisory Committee has stratified HM into myeloid neoplasm (MN), lymphoid neoplasm(LN), histiocytic neoplasm (HN), and mast cell disorders, mainly according to lineage origins [11]–[13]. HM originating from different lineages may be associated with different NI attack rates, due to the varying risk factors involved during disease progression. Most surveillance testing has focused on identifying the etiological pathogens of NIs, or on identifying the risk factors for bone marrow transplant patients, children, and severe HM patients (e.g., acute myeloid leukemia [AML] and acute lymphoblastic leukemia [ALL] patients) [1], [14]–[16]. For example, Huoi et al. have compared the incidence of NI in AML and ALL, and found that the incidence was higher among AML patients [1]. However, few studies have compared the incidence among all MN and LN patients. Therefore, establishing the relationship will hopefully facilitate the identification of patients at high risk of infection, and therefore provide an epidemiological basis for the development of targeted preventative strategies and control programs [17], [18]. In this study, we compared the incidence of NI among all MN and LN admissions, and applied multivariable logistic regression analysis to control for confounding effects.

## Methods

### Study population and case definition

We conducted a prospective surveillance of NIs among adult patients with HM, who were admitted to the Hematology Department at the General Hospital of the People's Liberation Army (PLAGH) for >48 hours between April 2010 and April 2013. PLAGH is an academic tertiary hospital with 3,600 beds, and 120,000 hospital admissions per year. The Hematology Department at PLAGH has 3 common wards with 96 beds, and a bone marrow transplant ward with 20 beds.

NI cases were mainly diagnosed according to the Center for Disease Control definition of NIs [1], [19], [20], and confirmation of NI was based on two principles. First, a combination of clinical findings, laboratory results, and other diagnostic tests was necessary for the diagnosis of NI. Second, for an infection to be considered nosocomial, it must have occurred >48 hours after patient admission, and there should be no evidence that the infection was present, or incubating, at the time of admission. Types of NI included nosocomial bacteremia, pneumonia, and urinary tract infections.

### Data collection

Our model included 10 covariates, and the expected proportion of positive cases in the population was 10%. Based on the work of Peduzzi et al. [21], the minimum number of admissions was calculated to be 1,000. We collected information regarding demographic characteristics (age, sex, date of admission and discharge) and clinical information, which included lineage of HM, current stem cell transplant status, neutropenia, chemotherapy, central venous catheterization (CVC), and infection status at admission. These factors were pre-determined prior to our analysis.

We also designed an electronic database, into which the resident physician entered the patient's information, including demographic characteristics, clinical information, and NI information. The electronic database also provided a communication platform, which enabled co-diagnosis by the resident physician and infection control professionals. Data were drawn from the electronic database by one trained physician, using a standardized format, and entered into an EpiData database (EpiData Association, Denmark). The data were further reviewed for accuracy and consistency by independent epidemiologists.

The research protocol was approved by the Ethics Committee of PLAGH, and written informed consent was obtained from the patients. Our research protocol did not harm patients' health, safety, or privacy.

### Statistical analysis

Descriptive statistics were evaluated; continuous variables were reported as mean and standard deviation, or median and interquartile range, while categorical variables were reported as frequencies and percentages. The NI attack rate was calculated as the number of NI admissions per 100 admissions, and the NI incidence was calculated as the number of NI episodes per 1,000 patients-days. Chi-square tests were used to compare NI attack rates. Stepwise logistic regressions were used to determine the independent risk factors (P<0.3 included, while P>0.1 excluded). Results were expressed as means and ranges, with 95% confidence intervals (CI). A two-sided P-value of <0.05 was considered statistically significant. The method of multiple comparisons for attack rates and percentages was verified using the Bonferroni test, and a nominal significance level less than 0.05/C^2^_n_ was considered statistically significant. All analyses were performed using SAS software, version 9.3.0 (SAS Institute, Cary, North Carolina).

## Results

### NI attack rates among malignancies of different lineages

**Figure 1:**
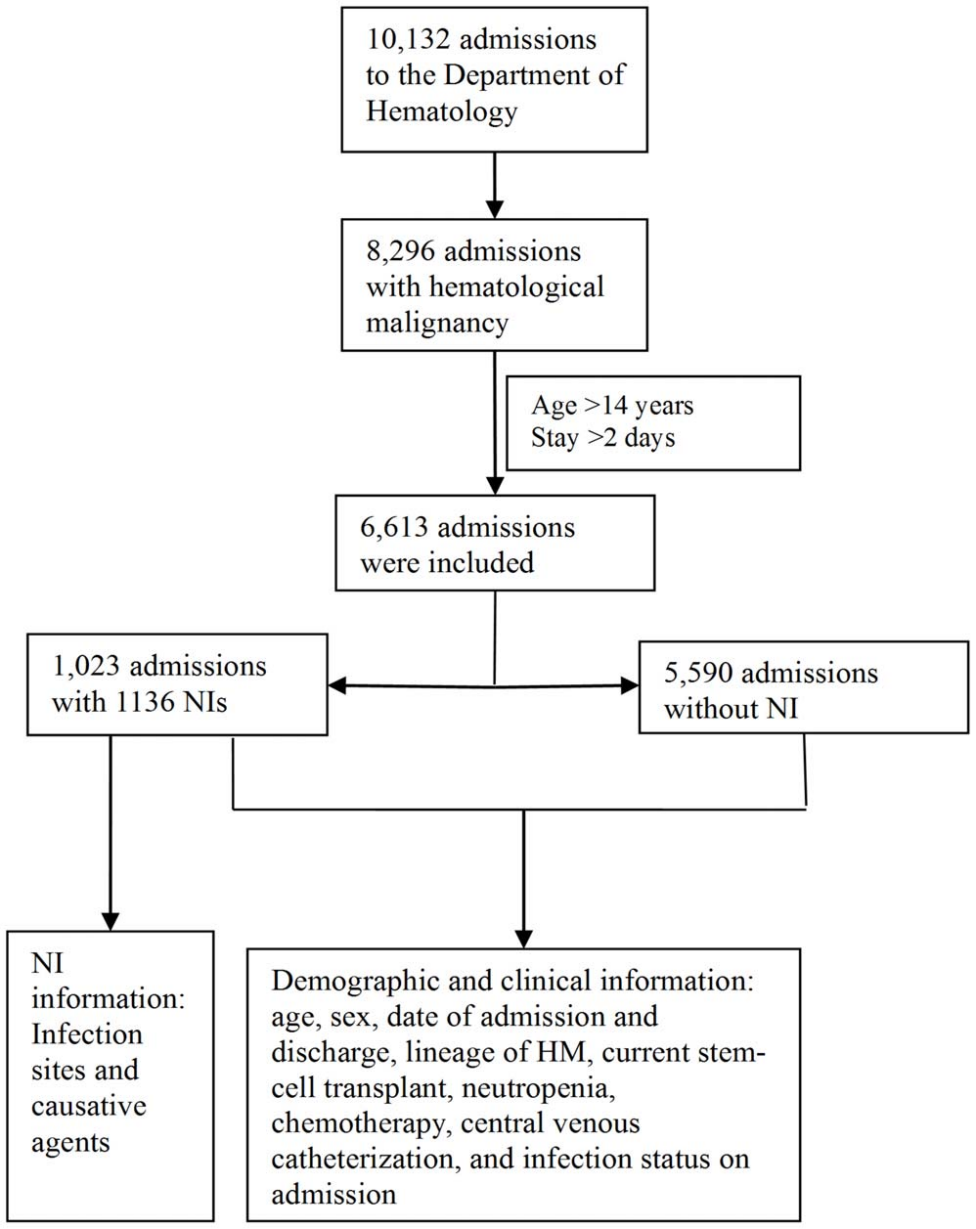
Flow diagram for the selection of eligible admissions and information collection.

During the study period, 6,613 admissions from 1,922 adult HM patients were included. Figure 1 describes the selection of eligible admissions and information collection. Patients averaged 3.44 admissions and hospital stays of 65.51 days. The mean age of the patients was 48.±17.56 years (range, 15–90 years), and 1,188 (61.81%) were male. Among HM, LN was the most common type (1,353, 70.40%), followed by MN (559, 29.08%) and HN (10, 0.52%). No mast cell disorders were observed (Table S1). A total of 1,023 (15.47%) admissions acquired 1,136 NI episodes, with an incidence of 9.6‰ (95% CI: 9.1–10.2). Among these episodes, 592 (52.20%) were pneumonia, 426 (37.57%) were bacteremia, and 116 (10.23%) were urinary tract infection. Seven hundred and eighty-five agents were isolated, of which 624 (79.49%) were bacteria and 161 (20.51%) were fungus. Four hundred and seventeen (66.83%) of the bacterial isolates were gram-negative and 207 (33.17%) were gram-positive.

Different NI attack rates were observed among HM from different lineages, with both the attack rate and incidence in MN lineages higher than those in LN lineages (28.42% vs. 11.00%, P<0.001 and 11.4‰ vs. 8.4‰, P<0.001). The NI attack rate and incidence for AML and myelodysplastic/myeloproliferative neoplasm (MDS/MPN) were higher than those for MDS (30.69% vs. 20.19%, P<0.001 and 38.89% vs. 20.19%, P = 0.003). The NI attack rates for T/NK-cell neoplasm and B-cell neoplasm were higher than those for Hodgkin lymphoma (15.04% vs. 3.65% and 10.94% vs. 3.65%, P<0.001; Table 1).

**Table 1:** Nosocomial infection (NI) among admissions with hematological malignancies from different lineages.

Origin of lineage	N*	NI attack rate#		D(d) ‡	NI incidence& (95% CI)	
		n†	%		m§	‰
Myeloid neoplasm:	1,717	488	28.42	47,391 (27.60)	542	11.4 (10.5–12.4)
Acute myeloid leukemia	1,196	367	30.69	33,486 (28.00)	405	12.1 (10.9–13.3)
Myeloproliferative neoplasm	155	37	23.87	4,461 (28.78)	39	8.7 (6.0–11.5)
Myelodysplastic syndrome	312	63	20.19	7,813 (25.04)	73	9.3 (7.2–11.5)
MDS/MPN¶	54	21	38.89	1,632 (30.22)	25	15.3 (9.4–21.3)
Lymphoid neoplasm:	4,853	534	11.00	70,421 (14.51)	592	8.4 (7.7–9.1)
T/NK-cell neoplasm	758	114	15.04	13,558 (17.89)	125	9.2 (7.6–10.8)
B-cell neoplasm	3,711	406	10.94	53,477 (14.41)	451	8.4 (7.7–9.2)
Hodgkin lymphoma	384	14	3.65	3,385 (8.82)	16	4.7 (2.4–7.0)
Histiocytic neoplasm	43	1	2.33	417 (9.70)	2	4.8 (0.6–17.2)
Total	6,613	1,023	15.47	118,229 (17.88)	1136	9.6 (9.1–10.2)

*Number of admissions;

† Number of admissions developing NI;

‡ Cumulative and average patient-days;

§ Number of NI episodes;

¶ Myelodysplastic/myeloproliferative neoplasm;

# Number of NI admissions per 100 admissions;

& Number of NI episodes per 1,000 patients-days.

### Risk factors associated with NI

Although the average age and admission frequency of LN admissions were higher than those of MN, the latter were more likely to have neutropenia, current stem cell transplant, infection on admission, and longer hospital stays (Table 2). The number of readmissions to the Hematology Department had no significant association with the incidence of NI. In both the univariate and multivariable regression, the presence of infection on admission was a risk factor associated with NI. Multivariable regression analysis also revealed that prolonged hospitalization, presence of CVC, neutropenia, current stem cell transplant, and old age were independently associated with a higher incidence of NI. After controlling for the effects of these factors, MN admissions still had a higher risk of infection compared to LN admissions (odds ratio: 1.34, 95% CI: 1.13–1.59, P<0.001; Table 3).

**Table 2:** Demographic and clinical characteristics of admissions with myeloid neoplasm and lymphoid neoplasm.

Variable	Myeloid neoplasm	Lymphoid neoplasm	P
Age (years)	42.48±17.11	48.68±17.69	<0.001
Male	1,027 (59.81)	3,044 (62.72)	0.003
Readmissions	3.27±2.73	3.42±2.60	0.032
Chemotherapy	1,233 (71.81)	3,908 (80.53)	<0.001
CVC	1,282 (74.67)	3,962 (81.64)	<0.001
Neutropenia	1,272 (74.08)	2,105 (43.38)	<0.001
Current stem cell transplant	200 (11.65)	148 (3.05)	<0.001
Infection on admission	329 (19.16)	399 (8.22)	<0.001
Length of hospital stay (days)	24.95 (12.30–35.18)	9.03 (5.06–18.98)	<0.001

CVC: central venous catheterization. Data presented as number (%), mean ± SD, or median (interquartile range), as appropriate.

**Table 3:** Unadjusted and adjusted risk factors for the development of nosocomial infection among myeloid neoplasm and lymphoid neoplasm admissions.

Variables	Unadjusted		Adjusted	
	OR (95% CI)	P	OR (95% CI)	P
Age	0.99 (0.986–0.993)	<0.001	1.01 (1.00–1.01)	0.033
Sex				
Male/Female	0.96 (0.84–1.10)	0.542		
Readmission number	0.98 (0.95–1.01)	0.118		
Length of hospital stay	1.09 (1.07–1.08)	<0.001	1.06 (1.06–1.07)	<0.001
Infection on admission				
Yes/No	2.43 (2.04–2.90)	<0.001	1.46 (1.18–1.80)	<0.001
Lineage origins				
Lymphoid neoplasm	Reference		Reference	
Myeloid neoplasm	3.21 (2.80–3.69)	<0.001	1.34 (1.13–1.59)	<0.001
Chemotherapy				
Yes/No	1.34 (1.14–1.60)	<0.001		
CVC				
Yes/No	1.73 (1.43–2.09)	<0.001	1.32 (1.07–1.63)	0.011
Neutropenia				
Yes/No	5.52 (4.66–6.54)	<0.001	2.35 (1.94–2.85)	<0.001
Current stem cell transplant				
Yes/No	13.17 (10.43–16.64)	<0.001	2.00 (1.50–2.66)	<0.001

CVC: central venous catheterization.

## Discussion

Although Huoi et al. have compared the incidence of NI in AML and ALL [1], there are few studies that have compared the incidence among all MN and LN patients, or identified the relationship between NI incidence and lineage. In our study, we compared NI attack rates, and identified independent risk factors in patients with HM originating from different lineages.

The overall NI attack rate in this study was 15.47% and the incidence was 9.6‰ (95% CI: 9.1–10.2), which are lower than previously reported values [1], [22]–[25]. Although most studies have focused on patients with stem cell transplants or neutropenia, the overall NI attack rate ranges from 40% to 50%, and the incidence ranges from 25‰ to 47‰ [22]–[25]. In addition, Huoi et al.'s study of HM found that the overall NI attack rate was 31.4% and the incidence was 18.2‰ (95% CI: 17.1–19.3) [1]. These differences may be explained by the fact that we included all HM patients (including those with leukemia or lymphoma), while most previous studies have included only severe patients with neutropenia and stem cell transplants. Lymphoma, especially Hodgkin lymphoma, typically occurs in localized regions and damages the immune system less than leukemia does, which might reduce the likelihood of contracting an NI. In addition, patients with HM were regularly re-admitted to the Department of Hematology for treatment. However, the patients' clinical conditions and treatment strategies (e.g., current stem cell transplant status, neutropenia, chemotherapy, CVC, and infection status) are unique for each admission. The risk of NI incidence for each admission is also unique, and it would be inappropriate to only consider one admission and infection in our analysis. Therefore, we calculated the attack rate based on admissions, rather than patients, which would inevitably lead to a lower incidence. Based on these reasons, the differences in NI incidence from our study and previous studies are acceptable.

Although our HM patients were frequently readmitted to the Hematology Department, the number of readmissions had no significant association with incidence of NI. Admissions with infection at admission, regardless of whether it was acquired in the community or at a previous admission, were at increased risk of NI. This could be caused by impaired function of the skin and mucous membrane system as a protective barrier against the causative microorganisms.

We did observe different NI attack rates among patients with HM originating from different lineages, indicating variability in each patient's vulnerability to NI. MN admissions had a higher NI attack rate compared to LN admissions, which is likely due to the higher frequency of neutropenia, current stem cell transplant, infection on admission, and longer hospital stay that is associated with this lineage. The presence of neutropenia, current stem cell transplant, infection on admission, and longer hospital stay have all previously been shown to be risk factors and predictors for NI in HM patients [10], [16], [26]–[28]. However, this trend remained after adjusting for these confounding factors, indicating that the differences in the immunological and biological mechanisms of the MN lineages also affected the vulnerability to NI. MN lineages have impaired differentiation and maturation of myeloid stem cells, including neutrophils, macrophages, and megakaryocytes [29], which are the basis of the innate immune system, and the first line of defense against bacterial and fungal infection [2], [11], [12]. In contrast, LN results in fewer mature T/NK and B-cells, and a reduced adaptive immune response, including cellular immunity and humoral immunity [2], [12], [13], although the innate system remains relatively intact to protect against NIs.

We also found that the risk of NI incidence in AML and MDS/MPN was higher than that in MDS. The underlying mechanism may be related to the fact that AML patients have more blast cells, but fewer mature myeloid cells [12]. In contrast, MDS is typically accompanied by an increase in the number of myeloblasts in the peripheral blood and bone marrow, although this increase is <20%, which is below the requisite threshold for a diagnosis of AML [30]. MDS/MPN includes cases that are inherently proliferative, and also show dysplastic features, including chronic myelomonocytic leukemia, atypical chronic myelogenous leukemia, and juvenile myelomonocytic leukemia. These diseases are characterized by many common features of MPN and MDS, including abnormalities in both granulocytic and monocytic lines, as well as a relative aggressive course that distinguish them from MDS [29]. The differences in lineage may contribute to the differences in susceptibility to NI between MDS/MPN and MDS. The NI attack rates in patients with T/NK-cell neoplasm or B-cell neoplasm were also higher than those in patients with Hodgkin lymphoma. As Hodgkin lymphomas typically arise in local lymph nodes, preferentially in the cervical region, they typically damage the immune system less than T/NKs and B-cell lymphomas [12], [30], and this effect may be responsible for the lower incidence of NI.

Our study had some limitations. First, HM originating from different lineages are often treated with unique chemotherapy schemes. Although we did consider several variables that were closely associated with the chemotherapy schemes (e.g., application of chemotherapy, current stem cell transplant, and neutropenia) in the multivariable logistic regression, the confounding effect of the chemotherapy schemes might still exist. Second, the stage of the disease (first line, progression, relapse), which might potentially affect NI incidence, was not included in our data collection. Finally, due to economic restrictions and traditional Chinese values, many critical and dying patients prefer to forego treatment and return home to await death, rather than die in-hospital. As we were unable to follow-up with these patients, the date of discharge or in-hospital death was considered the study endpoint. Therefore, we did not include mortality as a patient outcome. Even so, our hospital has one of the largest adult hematology departments in China, where patients throughout the country seek medical care, which ensures that our results are representative of HM cases. The prospective nature of the study, large sample size, single-center institution, and the extended duration of observation all ensure we have adequate statistical power to compare the incidence of NIs.

Based on our results, among patients with HM, special attention should be paid to MN admissions, especially those with AML and MDS/MPN, as these are at the highest risk of developing NIs. Education of healthcare providers who are involved in the day-to-day care of high-risk patients, as well as reallocation of medical resources, may help reduce the incidence of NIs. However, especially in developing countries with large populations, medical resources are limited and effective methods are needed to curb the incidence of NIs.

## Supporting Information

Table S1 **Demographic characteristics among patients with hematological malignancies originating from different lineages.**
(DOCX)
