# Supplementary material for: Nosocomial Infection in Adult Admissions with Hematological Malignancies Originating from Different Lineages: A Prospective Observational Study
Source: PLoS One. 2014 Nov 21;9(11):e113506. doi: 10.1371/journal.pone.0113506 (PMC4240653; doi:10.1371/journal.pone.0113506)
Supplement: Table S1 — Demographic characteristics among patients with hematological malignancies originating from different lineages. (DOCX) [file pone.0113506.s001.docx]

**Table S1.** Demographic characteristics among patients with hematological malignancies originating from different lineages

| Lineages | Patients(%)* | Admissions(Avg)^†^ | Hospital stay(day)^‡^ | Age(Avg±SD) | Male n(%) |
| --- | --- | --- | --- | --- | --- |
| Myeloid neoplasm | 559(29.08) | 1,717(3.07) | 47,391(84.78) | 45.42±17.52 | 335(59.93) |
| Acute myeloid leukemia | 331(59.21) | 1,196(3.61) | 33,486(101.17) | 42.69±16.75 | 190(57.4) |
| Myeloproliferative | 78(13.95) | 155(1.99) | 4,461(57.19) | 42.67±16.7 | 49(62.82) |
| Myelodysplastic syndrome | 132(23.61) | 312(2.36) | 7,813(59.19) | 53.13±17.58 | 83(62.88) |
| MDS/MPN^§^ | 18(3.22) | 54(3.00) | 1,632(90.67) | 51.06±17.58 | 13(72.22) |
| Lymphoid neoplasm | 1,353(70.40) | 4,853(3.59) | 70,421(52.05) | 49.66±17.45 | 847(62.60) |
| T/NK-cell neoplasm | 225(16.63) | 758(3.37) | 13,558(60.26) | 44.87±18.09 | 152(67.56) |
| T/NK-cell neoplasm | 1,016(75.09) | 3,711(3.65) | 53,477(52.63) | 52.53±16.37 | 630(62.01) |
| Hodgkin lymphoma | 112(8.28) | 384(3.43) | 3,385(30.22) | 33.26±14.34 | 65(58.04) |
| Histiocytic neoplasm | 10(0.52) | 43(4.30) | 417(41.70) | 40±13.99 | 6(60.00) |
| Total | 1,922(100.00) | 6,613(3.44) | 118,229(61.51) | 48.38±17.56 | 1,188(61.81) |

^*^Number and percentage of patients with hematological malignancy; ^†^Total admission times and average admission times per case; ^‡^Total hospital stay, and average hospitalized days per case; ^§^Myelodysplastic/myeloproliferative neoplasm.
